# Treatment of Inverted Nipple with Subareolar Abscess: Usefulness of High-Resolution MRI for Preoperative Evaluation

**DOI:** 10.1155/2012/573079

**Published:** 2012-07-11

**Authors:** Sayaka Enomoto, Kyoichi Matsuzaki

**Affiliations:** ^1^Department of Plastic and Reconstructive Surgery, Kawasaki Municipal Tama Hospital, 1-30-37 Shukugawara, Tama-Ku, Kawasaki 214-8525, Japan; ^2^Department of Plastic and Reconstructive Surgery, St. Marianna University School of Medicine, 2-16-1 Sugao, Miyamae-Ku, Kawasaki 216-8511, Japan

## Abstract

*Background*. Inverted nipples with subareolar abscesses can recur due to insufficient resection. It is important to provide reliable curative treatment after determination of the extent of resection by preoperative imaging evaluation. *Methods*. Ten patients were treated for inverted nipples with subareolar abscess. Sonography and high-resolution MRI were used as preoperative imaging modalities. The endpoints of preoperative imaging evaluation were defined as the identification of the abscess site, isolated fistula site, and extent of inflammation. *Results*. In all patients, sonography confirmed the presence of abscesses but their locations could not be identified. Sonography could not confirm the presence of isolated fistula or inflammation. In contrast, high-resolution MRI not only confirmed the presence of abscesses but also revealed their positional relationships with the nipples. In addition, high-resolution MRI confirmed the presence of isolated fistulas and inflammation as well as revealed their positional relationships with the nipples. In all patients, no recurrence was observed, and satisfactory surgical results were obtained. *Conclusion*. High-resolution MRI is useful in determination of the extent of resection of subareolar abscess associated with inverted nipple.

## 1. Introduction

Inverted nipples are esthetically unacceptable for many young women. In addition, they can make breast feeding difficult and cause mastitis and repeated subareolar abscesses. Recurrent subareolar abscesses can lead to esthetic problems such as multiple scars and nipple and breast distortion. In addition, they can cause symptoms such as areolar pain and fistula, resulting in major interference in daily living [1]. In recurrent cases, surgery is more difficult compared with cases undergoing initial surgery. Therefore, it is important to provide reliable curative treatment by initial surgery. However, there are many recurrent cases due to incomplete treatment [2, 3]. Surgery should be performed after determination of the extent of resection by preoperative imaging evaluation. This method is considered useful to improve the treatment outcomes by enabling reliable and optimal resection of the affected area. In this paper, we report on preoperative high-resolution MRI which was useful in image-guided surgery for the treatment of inverted nipple with subareolar abscess.

## 2. Materials and Methods

Ten patients were treated for inverted nipples with subareolar abscesses. All patients were women and their mean age was 31 years (range: 22 to 51 years). Their follow-up periods were at least 7 months after surgery. Sonography and high-resolution MRI were used as preoperative imaging modalities. A 1.5-T Intera Master unit (Philips Medical Systems) with a microscopy coil was used for high-resolution MRI [4]. The endpoints were defined as the identification of the abscess site, isolated fistula site, and extent of inflammation.

**Figure 1:**
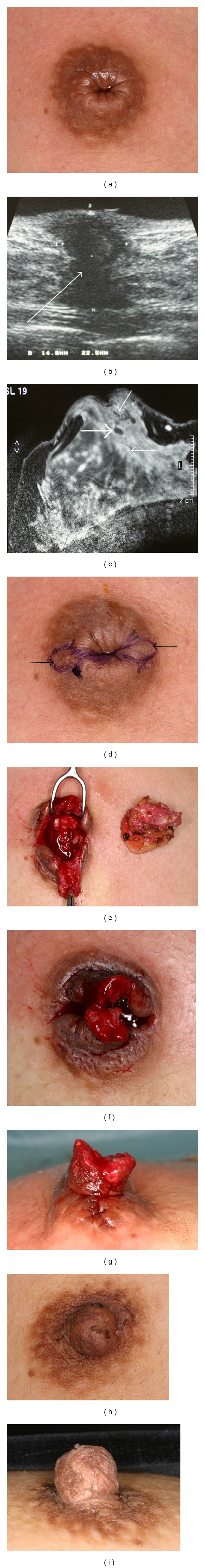
A 26-year-old woman. Inverted nipple with subareolar abscess (a). Sonography: the abscess was depicted as a hypoechoic area (arrow) (b). High-resolution MRI (contrast-enhanced T1-weighted image): abscess cavity (thick arrow) is hypointense structure with thin marginal enhancement. Small fistula (thin arrows) is hypointense linear structure associated with well enhanced inflammatory stroma (c). Surgical design. Dermal flaps were created at the neck of the nipple on both sides of the incision (arrows: before deepithelialization). The dermal flaps on both sides were marked for Z-plasty (d). Left: after excision, right: excised specimen (e). After completion of surgery, frontal aspect. The tip was not sutured to create roundness of the nipple (f). After completion of surgery, lateral aspect. The tip of the nipple was a raw surface (g). One year after surgery, frontal aspect. There was no recurrence of subareolar abscess (h). One year after surgery, lateral aspect. Since the neck of the rounded nipple was constricted, the nipple was less prone to reinvert (i).

**Figure 2:**
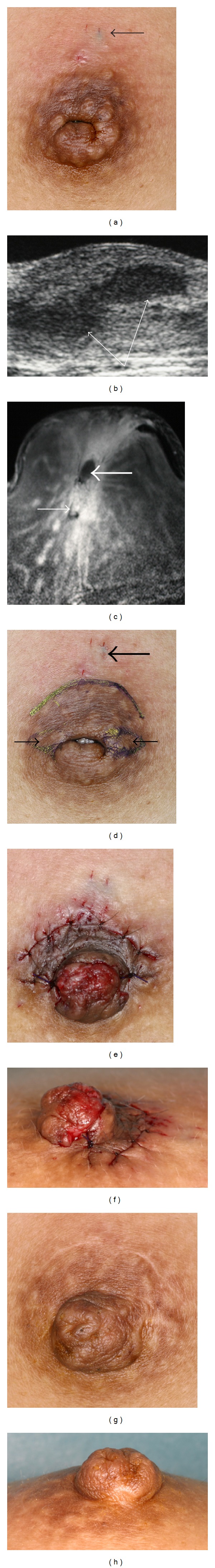
A 27-year-old woman. Inverted nipple with subareolar abscess. The abscess was extended subcutaneously beyond the areola and was stained blue by indigo carmine injection (arrow) (a). Sonography: the abscess was depicted as hypoechoic areas (arrows) (b). High-resolution MRI (Contrast-enhanced T1-weighted image): abscess cavity (thick arrow) is hypointense structure with thin marginal enhancement. Small fistula (thin arrow) is hypointense linear structure associated with well-enhanced inflammatory stroma (c). Surgical design. Dermal flaps were created at the neck of the nipple on both sides of the incision (thin arrows: before deepithelialization). The dermal flaps on both sides were marked for Z-plasty. The abscess extended beyond areola (thick arrow). Thus, an arc-like additional incision was made along the areolar margin and the affected area was reliably resected (d). After completion of surgery, frontal aspect. A portion of the nipple was not sutured and was left as a raw surface to create roundness of the nipple (e). After completion of surgery, lateral aspect (f). Seven months after surgery, frontal aspect. There was no recurrence of subareolar abscess (g). Seven months after surgery, lateral aspect. Since the neck of the rounded nipple was constricted, the nipple was less prone to reinvert (h).

In surgery, a subareolar abscess was stained by indigo carmine injection into the fistula. If the abscess was within the areola, then the causative lactiferous duct and abscess were excised en bloc through an incision, including the fistula in the inverted nipple area (Figures 1(a), 1(d), and 1(e)). If the abscess was beyond the areola, an arc-like incision was also made along the areolar margin to reliably resect the affected area (Figures 2(a) and 2(d)). Next, according to the method of Sakai et al. [5], the contracted scar tissue causing nipple inversion was detached through the incision of the nipple and was expanded. Subsequently, dermal flaps were created at the neck of the nipple on both sides of the incision (Figures 1(d) and 2(d)). The skin was gathered together around the dermal flaps and sutured. This procedure prevented nipple reinversion in the dead space formed after abscess excision. Z-plasty was performed at the sites where dermal flaps were created, and the neck of the nipple was constricted to further prevent reinversion. Roundness of the nipple was created by leaving a raw surface without suturing a portion of the lateral surface of the nipple. Petroleum jelly was used to stimulate epithelialization (Figures 1(f), 1(g), 2(e), and 2(f)).

## 3. Results

 In all patients, sonography confirmed the presence of abscesses but their locations could not be identified. Sonography could not confirm the presence of isolated fistula or inflammation (Figures 1(b) and 2(b)). In contrast, high-resolution MRI not only confirmed the presence of abscesses but also revealed their positional relationships with the nipples. In addition, high-resolution MRI confirmed the presence of isolated fistulas and inflammation as well as revealed their positional relationships with the nipples (Figures 1(c) and 2(c)) (Table 1). This imaging modality enabled preoperative evaluation of the lesions in the nipple and subareolar area. Thus, it was useful in determination of the extent of resection. In all patients, no recurrence was observed and satisfactory surgical results were obtained (Figures 1(h), 1(i), 2(g), and 2(h)).

## 4. Discussion

 Inverted nipples with subareolar abscesses often have severe inversion due to scar tissue from inflammation. It is important to properly treat inverted nipples by surgery to prevent recurrence of subareolar abscess. In such a surgery, considerations need to be made to prevent reinversion of the newly formed nipple in the dead space, created by the resection in the abscess area. In this study, the method of Sakai et al. [5] was followed and reinversion was prevented in all patients.

 Subareolar abscesses can recur due to insufficient resection. In recurrent cases, surgery is difficult compared with cases undergoing initial surgery. Therefore, it is important to provide reliable curative treatment for subareolar abscess by initial surgery. This point is illustrated by the use of mastectomy with oncoplastic techniques for recurrent abscess cases [3]. The breast surgeon Lannin [6] reported on the results of treatment that he performed for subareolar abscesses in a 22-year period. Approximately half of the 67 cases that he experienced were managed medically. In the remaining half, radical elliptical incision with primary closure resulted in low long-term recurrence rates. Although stable treatment outcomes have been reported by such an experienced surgeon, there are still many cases of recurrent subareolar abscesses due to incomplete treatment [1–3, 6]. Thus, surgery should be performed after determination of the extent of resection by preoperative imaging evaluation. This method is considered useful to improve treatment outcomes by enabling reliable and optimal resection of the affected area. In particular, in postgraduate education in teaching hospitals, it is important to perform image-guided surgery using preoperative imaging data and to improve the ability to diagnose through postoperative review of surgical findings.

 Sonography is an imaging modality prevalently used in the evaluation of subareolar abscess. Fu et al. [4] reported that 83% of the patients with a history of recurrent subareolar abscesses showed sonographic findings suggestive of abscess or fistula. They stated that sonography did not reveal the lesions if the patients had isolated fistulas, particularly for fistulas inside the nipples. In contrast, high-resolution MRI enabled the detection of a 1.5 mm fistula in the nipple [4]. In our study, sonography confirmed the presence of abscess cavities in all 10 patients, but small lesions were undetectable. High-resolution MRI identified the locations of the abscess, isolated fistula, and inflammation. In addition, this imaging modality enabled the confirmation of the extent of these pathologies and their positional relationships with the inverted nipple from the depicted shape of the nipple. Thus, our results suggest that high-resolution MRI is a useful preoperative imaging modality.

 Sonography is an important, noninvasive medical tool. However, sometimes it causes strong pain when a probe is placed on the affected area with severe inflammation from subareolar abscess. MRI is a good diagnostic imaging tool that does not cause radiation damage like computed tomography. However, injections of gadodiamide hydrate are used for high-resolution MRI. Thus, caution is required because high-resolution MRI cannot be used in cases in which contrast agent administration is contraindicated.

## 5. Conclusion

 High-resolution MRI is useful in determination of the extent of resection of subareolar abscess associated with inverted nipple.

## Figures and Tables

**Table 1:** Findings of high-resolution MRI and sonography.

Imaging findings	High-resolution MRI	Sonography
Abscess cavity	Presence of abscess cavity confirmed and positional relationship with inverted nipple revealed	Presence of abscess cavity confirmed but the location not identifiable
Isolated fistula	Presence of isolated fistula confirmed and positional relationship with inverted nipple revealed	Undetectable
Inflammatory signs	Presence of inflammation confirmed and positional relationship with inverted nipple revealed	Undetectable
